# Letter to the Editor regarding “Effect of ICU quality control indicators on VAP incidence rate and mortality: a retrospective study of 1267 hospitals in China”

**DOI:** 10.1186/s13054-023-04324-w

**Published:** 2023-01-20

**Authors:** Mingqiang Wang, Lingtong Huang, Xiaohan Huang

**Affiliations:** 1 Department of Critical Care Medicine, Zhengzhou University People’s Hospital, Henan University People’s Hospital, Zhengzhou, People’s Republic of China; 2 Department of Critical Care Medicine, Henan Key Laboratory for Critical Care Medicine, Zhengzhou Key Laboratory for Critical Care Medicine, Henan Provincial People’s Hospital, Zhengzhou, People’s Republic of China; 3 Department of Critical Care Units, The First Affiliated Hospital, Zhejiang University School of Medicine, Hangzhou, People’s Republic of China; 4 Department of Nephrology, The First Affiliated Hospital, Zhejiang University School of Medicine, Hangzhou, People’s Republic of China

Dear Editor,

We read the article by Xin et al. [1] published on Critical Care with great interest. This study includes 1,091,878 patients from 1267 hospitals. The authors analyzed the quality control indicators and found that the structural factors were associated with the incidence of ventilator-associated pneumonia (VAP), but not with VAP mortality. VAP incidence and mortality were associated with the rate of unplanned endotracheal extubation and reintubation within 48 h. However, some issues in this study remain unresolved.

First of all, the transfer of patients between intensive care units (ICUs) is common in China. Patients may transfer from secondary or tertiary hospitals to teaching hospitals in search of better medical care. Additionally, stable patients will be transferred from the intensive care units of teaching hospitals to those of secondary hospitals. It’s necessary to clarify whether the ventilator days of the patients who transferred between hospitals with endotracheal intubation were properly estimated.

Secondly, we regret that Xin et al. did not undertake a subgroup analysis based on specialized intensive care units and different hospital levels in this study as described before [2], which could have had a substantial impact on the main findings. Patient management varies between specialized intensive care units. As described by Xin et al. [1] in this article, the majority of intensive care unit (ICU) physicians come from other disciplines. Cardiologists who work in Cardiac Care Units and Respiratory Doctors who work in Respiratory Intensive Care Units can be found in numerous Chinese hospitals. And the diverse backgrounds of doctors in these specialized ICUs may result in varying VAP incidence and mortality rates. Although the heterogeneity of different specialized intensive care units can be improved through unified training, it is currently unavoidable. Due to the lack of appropriate analysis, the study by Xin et al. may conceal certain facts.

Finally, Xin et al. only gave data from Poisson regression analysis, with no adjustment for age, gender, or comorbidities and Bayesian logistic regression models adjusted for these factors at baseline may be more suitable for this study.

Overall, this is a crucial study for ICU development in China; however, the methodology may have significant impacts on the main findings. If the authors are able to overcome the concerns we've raised, this study may contribute to the further improvement of quality control in Chinese ICUs.

## Data Availability

None.
